# A serial optical frequency-domain imaging study of early and late vascular responses to bioresorbable-polymer sirolimus-eluting stents for the treatment of acute myocardial infarction and stable coronary artery disease patients: results of the MECHANISM-ULTIMASTER study

**DOI:** 10.1007/s12928-021-00777-4

**Published:** 2021-04-25

**Authors:** Tomonori Itoh, Hiromasa Otake, Takumi Kimura, Yoshiro Tsukiyama, Tatsuo Kikuchi, Munenori Okubo, Takatoshi Hayashi, Takayuki Okamura, Shoichi Kuramitsu, Takashi Morita, Shinjo Sonoda, Shozo Ishihara, Nehiro Kuriyama, Takaaki Isshiki, Tsunenari Soeda, Kiyoshi Hibi, Toshiro Shinke, Yoshihiro Morino, the MECHANISM-ULTIMASTER study group, Yudai Shimoda, Takumi Inoue, Hiroyuki Okura, Takashi Takenaka, Masaki Sakakibara, Yasushi Jinno, Yoshinori Yasaka, Tomofumi Takaya, Junya Shite, Amane Kozuki, Makoto Kadotani, Yasuhiro Kaetsu, Yoshitomo Tsutsui, Mamoru Mochizuki, Naoki Masuda, Kengo Tanabe, Kengo Tanabe, Hideki Ishii, Yoritaka Otsuka, Mitsuru Abe

**Affiliations:** 1 Division of Cardiology, Department of Internal Medicine, Iwate Medical University, 2-1-1, Idai-Dori, Yahaba-Cho, Siwa-Gun, Yahaba, Iwate 028-3695 Japan; 2 Division of Cardiology, Department of Internal Medicine, Graduate School of Medicine, Kobe University, Kobe, Japan; 3 Division of Cardiology, Edogawa Hospital, Edogawa, Tokyo, Japan; 4 Department of Cardiovascular Medicine, Gifu Heart Center, Gifu, Japan; 5 Department of Cardiology, Hyogo Prefectural Awaji Medical Center, Sumoto, Hyogo Japan; 6 Department of Medicine and Clinical Science, Yamaguchi University Graduate School of Medicine, Ube, Japan; 7 Department of Cardiology, Kokura Memorial Hospital, Kitakyushu, Japan; 8 Division of Cardiology, Osaka General Medical Center, Osaka, Osaka Japan; 9 University of Occupational and Environmental Health, Kitakyusyu, Japan; 10 Mimihara General Hospital, Sakai, Osaka Japan; 11 Miyazaki Medical Association Hospital, Miyazaki, Miyazaki Japan; 12 Ageo Central General Hospital, Saitama, Japan; 13 Nara Medical University, Kashihara, Japan; 14 Division of Cardiology, Yokohama City University Medical Center, Yokohama, Kanagawa Japan; 15 Division of Cardiology, Department of Medicine, Showa University, Tokyo, Japan

**Keywords:** Bioresorbable-polymer sirolimus-eluting stents, ST-elevation myocardial infarction, Stable coronary artery disease, Optical frequency-domain images

## Abstract

The purpose of this study was to assess early and late vascular healing in response to bioresorbable-polymer sirolimus-eluting stents (BP-SESs) for the treatment of patients with ST-elevation myocardial infarction (STEMI) and stable coronary artery disease (CAD). A total of 106 patients with STEMI and 101 patients with stable-CAD were enrolled. Optical frequency-domain images were acquired at baseline, at 1- or 3-month follow-up, and at 12-month follow-up. In the STEMI and CAD cohorts, the percentage of uncovered struts (%US) was significantly and remarkably decreased during early two points and at 12-month (the STEMI cohort: 1-month: 18.75 ± 0.78%, 3-month: 10.19 ± 0.77%, 12-month: 1.80 ± 0.72%; *p* < 0.001, the CAD cohort: 1-month: 9.44 ± 0.78%, 3-month: 7.78 ± 0.78%, 12-month: 1.07 ± 0.73%; *p* < 0.001 respectively). The average peri-strut low-intensity area (PLIA) score in the STEMI cohort was significantly decreased during follow-up period (1.90 ± 1.14, 1.18 ± 1.25, and 1.01 ± 0.72; *p* ≤ 0.001), whereas the one in the CAD cohort was not significantly changed (0.89 ± 1.24, 0.67 ± 1.07, and 0.64 ± 0.72; *p* = 0.59). In comparison with both groups, differences of %US and PLIA score at early two points were almost disappeared or close at 12 months. The strut-coverage and healing processes in the early phase after BP-SES implantation were significantly improved in both cohorts, especially markedly in STEMI patients. At 1 year, qualitatively and quantitatively consistent neointimal coverage was achieved in both pathogenetic groups.

**Supplementary Information:**

The online version contains supplementary material available at 10.1007/s12928-021-00777-4.

## Introduction

First-generation drug-eluting stents (DESs) have achieved remarkable results [1], lowering the 1-year restenosis rate to less than 10%. Although these stents inhibit the excess intimal proliferation that causes restenosis and delay the regeneration of the neointima [2], cases of late stent thrombosis have been reported [3]. Polymer coatings applied to the stent as drug carriers and permanent residence of the stent in the coronary vessels have been attracting attention as causes of persistent inflammation leading to stent thrombosis [4]. A second-generation drug-eluting stent, the cobalt–chromium everolimus-eluting stent (CoCr-EES), which was developed after these concerns were raised, showed early stage re-endothelialization of stent struts and reduction of inflammation compared to first-generation DESs [5]. As a result, several clinical studies showed a decrease in the incidence of stent thrombosis when CoCr-EESs rather than other DESs were used [6]. These results suggest that the polymer and drug used for the CoCr-EES have good antithrombogenicity [5]. However, since the polymers in the CoCr-EES remain in place, there are concerns regarding long-term problems during the follow-up period.

The Ultimaster^®^ stent is a new reduced-dose sirolimus-eluting stent that uses an abluminal bioabsorbable coating on a thin-strut platform (Terumo Corporation, Tokyo, Japan: BP-SES); this stent has the potential to allow arterial healing more firmly than the early generation of permanent-polymer-based DESs. Although bioabsorbable polymer-based drug-eluting stent technology may theoretically promise improved healing of treated segments [4], clinical trials to characterize their effects on the vessel in quantitative and qualitative terms remain limited. Moreover, the time course of early and convalescent vascular healing has not been fully elucidated in ST-elevation myocardial infarction (STEMI) or stable coronary artery disease (CAD), which hampers any understanding of the mechanisms relevant to risk reduction for stent thrombosis.

The purpose of this study was to assess early and late vascular healing in response to BP-SESs in the treatment of patients with STEMI and stable-CAD.

## Methods

### Study protocol (Fig. 1)

**Fig. 1:**
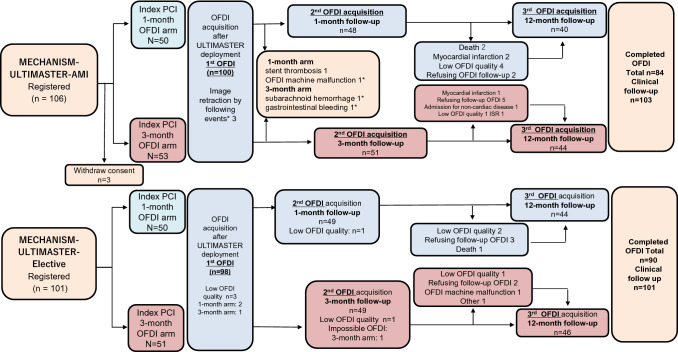
Study protocol and patient flowchart for this study

The MECHANISM-ULTIMASTER-AMI (STEMI cohort; UMIN 000021549) and MECHANISM-ULTIMASTER-Elective (stable-CAD cohort; UMIN 000021119) studies were prospective multicenter registries of BP-SES. A definition of the study abbreviation and a list of all participating institutes can be found in the appendix of the supplemental materials.

Patients were recruited between April 2016 and May 2017 from the participating institutions. The key inclusion criteria were as follows: (1) in the MECHANISM-ULTIMASTER-AMI study, STEMI patients who met the diagnostic criteria for the third universal definition of myocardial infarction, (2) in the MECHANISM-ULTIMASTER-Elective study, patients with stable-CAD that was diagnosed by an ischaemia-positive stress test (by an imaging or functional testing modality) or the presence of ischaemic chest pain (no change in the frequency, duration, or intensity of symptoms within 4 weeks). One lesion per patient was registered in each study.

The key exclusion criteria were a left main lesion for both registries and cardiogenic shock for the AMI registry. The patient inclusion and exclusion criteria for both studies are described in detail in the supplemental materials.

Coronary optical frequency-domain imaging (OFDI) was performed immediately after percutaneous coronary intervention (PCI) to confirm optimal stent expansion. In both cohorts, follow-up of index stent was performed either 1 month or 3 months after the index PCI during staged PCI for residual stenotic lesions or follow-up angiography (2nd OFDI: 1 month; 30 ± 10 days, 3 months; 90 ± 20 days), and another follow-up was performed at 12 months (3rd OFDI: 12 ± 2 months), completing the series of timepoints (Fig. 1). The LUNAWAVE® imaging system with the FastView^®^ imaging catheter (Terumo Corporation, Tokyo, Japan) was used in the present study. All quantitative coronary angiography (QCA) data were evaluated at Iwate Core Analysis Laboratory (ICAL) as the central core laboratory. The details of the PCI procedure, OFDI acquisition, and QCA protocol are described in the supplemental material. Assignment to 1-month or 3-month follow-up was determined at physicians’ discretion with their patients’ approval. Dual antiplatelet therapy was described in the supplemental file.

### OFDI image analysis and study endpoints

**Fig. 2:**
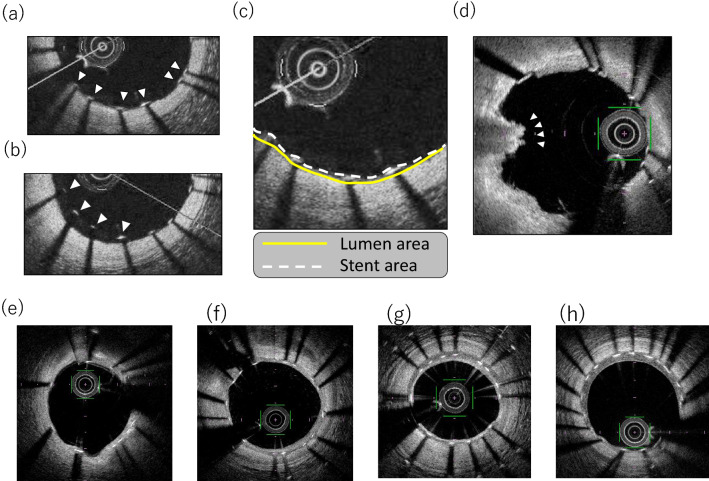
OFDI analysis: **a** uncovered struts; **b** malapposed struts; **c** stent and lumen area; **d** thrombus (a mass attached of the luminal surface stent strut or floating within the lumen); **e** PLIA score: Grade 0; **f** Grade 1; **g** Grade 2; **h** Grade 3

OFDI analysis was performed by two independent core laboratories (ULTIMASTER AMI: Kobe Cardiovascular Core Laboratory (KCCL), Kobe University Graduate School of Medicine, Kobe, Japan, MECHANISM-ULTIMASTER Elective: ICAL, Iwate Medical University, Yahaba, Japan) using OFDI software (Terumo, Japan) and according to the standard operating procedure of the MECHANISM-ULTIMASTER study and published methods (Fig. 2) [7–9]. Inter- and intra-observer reproducibility were demonstrated in supplemental file. Inter-core-laboratory reproducibilities were as follows: cover/uncovered: 0.80, PLIA score: 0.97.

### Post-stenting and follow-up OFDI

Quantitative OFDI analyses were performed at every 1-mm interval for the assessment of lumen, stent, and intra-stent tissue area (stent area—lumen area). Intra-stent tissue thickness was measured from the lumen border to the center of the strut blooming. Struts with intra-stent tissue thickness less than 0 μm were defined as uncovered struts. A strut that was partially covered with tissue was classified as an uncovered strut. A strut with a maximum distance of > 115 μm (metal thickness: 80 μm, coating thickness 15 μm, and axial resolution 20 μm) between its center reflection and the adjacent vessel surface was defined as a malapposed strut.

Qualitative OFDI analysis was performed for every individual frame to assess intra-stent tissue (intra-stent thrombus (> 0.25 mm), irregular protrusion (IRP; > 0.25 mm), smooth protrusion, and fibrous-tissue disruption). The definition of these intra-stent tissues was described in supplemental file. The peri-strut low-intensity area (PLIA) score was defined according to a previous method [8]. When PLIA was detected in at least one cross-section, PLIA was considered to be present in the patient. PLIA scores were defined as follows: score 1 for PLIA that proliferated only around the stent struts, 2 for PLIA observed in part of the neointima, and 3 for PLIA observed in the entire neointima (Fig. 2).

### Endpoints

#### Primary endpoint

The primary endpoint was the percentage of uncovered struts (%US) at 1 month for the STEMI cohort or at 3 months for the stable-CAD cohort as measured by OFDI analysis. Similarly, the %US at 3 months for the STEMI cohort and at 1 month for the stable-CAD cohort were assessed for supplementary statistical comparison.

#### Secondary endpoints

The secondary endpoints included (1) clinical endpoints and (2) OFDI parameters during the 12-month follow-up period. The clinical endpoints of interest were all-cause death, cardiac death, MI, stroke, and major bleeding within 12 months. The efficiency endpoints consisted of any target lesion revascularization (TLR), clinically driven TLR, any target vascular revascularization (TVR), aorto-coronary bypass grafting, any revascularization at 12 months, and angiographic binary restenosis at 12 months. Composite endpoints including device-oriented cardiac events (DOCEs) were described in supplemental file. The above endpoint defined for comparing the STEMI and stable-CAD cohorts was set post hoc.

### Statistical analysis

The rationale used to select the number of cases is described in the supplemental materials. The almost data are presented as the mean ± SD. We used a random-effects model to compare OFDI findings, such as %US, % malapposed struts, and neointimal thicknesses (data are presented as mean ± SE) between the STEMI and stable-CAD groups. The model included random effects at the level of cross-sections within groups and exchangeable serial correlations over time. The software used for multilevel analysis was SAS ver. 9.4 (SAS Institute Inc., Cary, NC, USA).

## Results

### Baseline characteristics of patients, lesions, and procedures

Figure 1 depicts the study protocol and a patient flowchart. Among 106 patients (106 lesions) enrolled in the STEMI cohort and 101 in the stable-CAD cohort (101 lesions), sufficient OFDI assessments were impossible in several cases for a variety of reasons, which are displayed in detail in Fig. 1. The baseline characteristics of the patients, lesions, and procedures for the study population are shown in Table 1. Both groups continued to receive dual antiplatelet therapy (DAPT), primarily with clopidogrel (Supplemental Table 1). Table 2 shows a baseline qualitative and quantitative measurement of OFDI findings in the two groups.

**Table 1:** Baseline clinical characteristics

Variable	STEMI (*n* = 103)	Stable-CAD (*n* = 101)
Age, years	66.2 ± 10.8	68.0 ± 9.6
Gender, male, *n* (%)	84 (81.6)	73 (72.3)
Diabetes mellitus, *n* (%)	51 (49.5)	47 (46.5)
Dyslipidaemia, *n* (%)	80 (77.7)	81 (80.3)
Hypertension, *n* (%)	71 (68.9)	81 (78.6)
Smoking, *n* (%)	64 (62.1)	46 (45.5)
Prior MI, *n* (%)	2 (1.9)	8 (7.9)
Prior PCI, *n* (%)	3 (2.9)	26 (25.7)
30 ≤ eGFR < 60, eGFR <30, *n* (%)	24/1 (23.3/0.97)	31/1 (31.3/1.0)
Peak CK value (IU/L)	1721 (79-13916)	91 (22-304)
Medication at index PCI		
DAPT, *n* (%)	103 (100)	100 (99.0)
Aspirin, *n* (%)	103 (100)	100 (99.0)
Thienopyridine, *n* (%)	103 (100)	101 (100)
Statins, *n* (%)	98 (95.1)	86 (85.1)
ACE/ARB, *n* (%)	94 (91.3)	53 (52.5)
β-blockers, *n* (%)	86 (83.5)	46 (45.5)
Lesion characteristics		
LAD, LCX, RCA, *n* (%)	28 (27.2)/17 (16.5)/58 (56.3)	37 (36.6)/22 (21.8)/42 (41.6)
ACC/AHA classification (A/B1/B2/C)	0/7/30/64	0/16/16/69
Postprocedural QCA and stent use		
Prox. reference diameter, mm	3.26 ± 0.53	3.21 ± 0.48
Dist. reference diameter, mm	2.73 ± 0.61	2.63 ± 0.49
Minimum lumen diameter (mm)	2.57 ± 0.45	2.58 ± 0.44
% diameter stenosis (%)	13.69 ± 7.82	11.13 ± 10.13
Number of stents used	1.21 ± 0.41	1.20 ± 0.45
Diameter of stent used (mm)	3.23 ± 0.69	3.02 ± 0.65
Total stent length (mm)	29.63 ± 13.22	30.14 ± 14.28

*PCI* percutaneous coronary intervention, *MI* myocardial infarction, *GFR* glomerular filtration rate (/min./1.73m^2^), *CK* creatinine kinase, *DAPT* dual antiplatelet therapy, *ACE* angiotensin converting enzyme inhibitor, *ARB* angiotensin II receptor blocker, *LAD* left anterior descending artery, *LCx* left circumflex artery, *RCA* right coronary artery

**Table 2:** Baseline OFDI data: qualitative and quantitative measurement of OFDI at postprocedural PCI

Variable	STEMI (*n* = 100)	Stable-CAD (*n* = 96)
Lesion level analysis	(*n* = 100)	(*n* = 96)
Stent length (mm)	28.68 ± 12.70	26.94 ± 10.69
Average stent area (mm^2^)	7.20 ± 2.02	6.84 ±1.92
Minimal stent area (mm^2^)	5.91 ± 1.90	5.67 ± 1.85
Maximal stent area (mm^2^)	8.36 ± 2.49	7.94 ± 2.16
Relative stent expansion	1.04 ± 0.45	0.96 ± 0.39
Cross-section level analysis	(*n* = 2953)	(*n* = 2777)
Number of frames per lesion	29.5±12.4	28.3±10.7
Average lumen area (mm^2^)	7.09±0.19^a^	6.92±0.19^a^
Average stent area (mm^2^)	7.22±0.20^a^	6.84±0.20^a^
Average malapposed area (mm^2^)	0.288±0.020^a^	0.320±0.016^a^
Stent strut level analysis	(*n* = 31,605)	(*n* = 28,640)
Number of struts per lesion	316.1±140.6	292.2±120.8
Number of struts per frame	10.7±3.0	10.3±3.1
% malapposed strut (%)	4.87±0.37^a^	4.88±0.35^a^
% uncovered strut (%)	65.07±1.73^a^	79.88±1.75^a^
Qualitative lesion level analysis	(*n* = 100)	(*n* = 96)
Dominant postprocedural IST characteristics (sp/dftp/ip/th)	NA	17/28/10/41 (17.7%/29.2%/10.4%/42.7%)
Thrombus > 0.25 mm (%)	NA	43 (44.8%)
ip > 0.25 mm (%)	NA	14 (14.6%)
Smooth protrusion > 0.25 mm (%)	NA	91 (94%)
Disrupted fibrous-tissue protrusion > 0.25 mm (%)	NA	70 (72.9%)
Any protrusion > 0.25 mm	100 (100%)	96 (100%)
Proximal edge dissection (%)	10 (10.0%)	21 (21.9%)
Distal edge dissection (%)	5 (5.0%)	13 (13.5%)

*NA* not available, *IST* intra-stent tissue, *sp* smooth protrusion, *dfp* disrupted fibrous-tissue protrusion, *ip* irregular protrusion, *th* thrombus

^a^Standard error

### Primary and other OFDI endpoints of the STEMI cohort

**Fig. 3:**
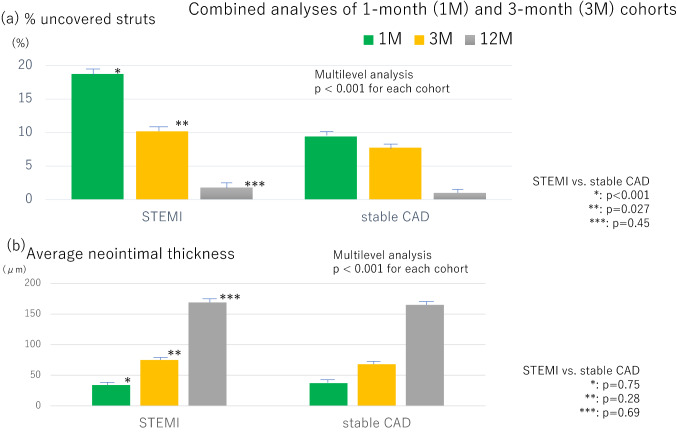
The percentage of uncovered struts (**a**) and averaged neointimal thickness (**b**) at each follow-up visit in the both groups

OFDI measurements were serially compared between the immediate post-intervention timepoint and either 1- or 3-month follow-up, as shown in Table 3. In the STEMI cohorts, the %US significantly decreased between these timepoints (18.72 ± 0.78% to 10.16 ± 0.77%), confirming prompt and remarkably progression of strut coverage by the first 3 months in Fig. 3 (a). Moreover, the progression of strut coverage was maintained for 12 months (%US at 12 months: 1.78 ± 0.73%; *p* < 0.001). In parallel, the thickness of the neointima (NIT) consistently increased during the 12 months of follow-up period as Fig. 3b.

**Table 3:** Quantitative and qualitative OFDI analysis: STEMI and stable-CAD cohorts

	STEMI cohort					Stable-CAD cohort				
	Post	1 month	3 months	12 months	*P *value	Post	1 month	3 months	12 months	*P* value
Quantitative lesion level analysis	(*n* = 100)	(*n* = 48)	(*n* = 51)	(*n* = 84)		(*n* = 98)	(*n* = 49)	(*n* = 49)	(*n* = 90)	
Minimum SA (mm^2^)	5.91 ± 0.19	6.05 ± 0.19	5.91 ± 0.19	6.05 ± 0.19	0.027	5.66 ± 0.19	5.73 ± 0.19	5.76 ± 0.19	5.74 ± 0.19	0.24
Minimum LA (mm^2^)	5.44 ± 0.18	5.70 ± 0.20	5.36 ± 0.19	4.26 ± 0.18	< 0.001	5.60 ± 0.18	5.51 ± 0.20	5.39 ± 0.20	4.27 ± 0.18	< 0.001
Cross-section base analysis	(*n* = 2953)	(*n* = 1367)	(*n* = 1565)	(*n* = 2411)		(*n* = 2777)	(*n* = 1401)	(*n* = 1312)	(*n* = 2500)	
No. of frames per lesion	29.5 ± 12.4	28.5 ± 13.4	30.7 ± 10.9	28.7 ± 11.7		28.3 ± 10.7	28.6 ± 10.7	26.8 ± 10.1	27.8 ± 10.6	
Lumen area (mm^2^)	7.08 ± 0.19	7.28 ± 0.19	6.94 ± 0.19	5.99 ± 0.19	< 0.001	6.95 ± 0.19	6.99 ± 0.19	6.85 ± 0.19	5.69 ± 0.19	< 0.001
Malapposed area (mm^2^)	0.287 ± 0.018	0.315 ± 0.021	0.280 ± 0.020	0.080 ± 0.019	< 0.001	0.321 ± 0.018	0.333 ± 0.020	0.288 ± 0.021	0.053 ± 0.019	< 0.001
Averaged NIT(μm)	NA	0.034 ± 0.004	0.075 ± 0.004	0.169 ± 0.004	< 0.001	NA	0.037 ± 0.004	0.069 ± 0.004	0.166 ± 0.003	< 0.001
Averaged NIA (mm^2^)	NA	0.307 ± 0.046	0.553 ± 0.046	1.395 ± 0.044	< 0.001	NA	0.241 ± 0.033	0.476 ± 0.000	1.297 ± 0.026	< 0.001
Strut level analysis	(*n* = 31,605)	(*n* = 14,477)	(*n* = 16,491)	(*n* = 24,928)		(*n* = 28,640)	(*n* = 14,779)	(*n* = 13,498)	(*n* = 25,685)	
Number of struts per lesion	316.1 ± 140.6	301.6 ± 153.5	323.4 ± 122.5	296.8 ± 119.5		292.2 ± 120.8	301.6 ± 122.5	275.5 ± 108.2	285.4 ± 116.0	
Number of struts per frame	10.7 ± 3.0	10.6 ± 3.1	10.5 ± 3.0	10.3 ± 3.0		10.3 ± 3.1	10.5 ± 3.2	10.3 ± 3.0	10.3 ± 3.2	
% Uncovered strut (%)	*N*A	18.72 ± 0.78	10.16 ± 0.77	1.78 ± 0.73	< 0.001	*N*A	9.44 ± 0.78	7.78 ± 0.78	1.07 ± 0.73	< 0.001
% Malapposed strut (%)	4.87 ± 0.33	2.68 ± 0.39	1.51 ± 0.38	0.60 ± 0.34	< 0.001	4.88 ± 0.33	3.29 ± 0.39	1.74 ± 0.39	0.09 ± 0.34	< 0.001
Qualitative OFDI assessment	(*n* = 100)	(*n* = 48)	(*n* = 51)	(*n* = 84)		(*n* = 98)	(*n* = 49)	(*n* = 49)	(*n* = 90)	
IRP (%)	NA	43.3 ± 1.3*	17.0 ± 1.5^†^	2.3 ± 2.1^‡^	< 0.001	14.3 ± 1.3	4.4 ± 2.0*	1.8 ± 2.9^†^	1.1 ± 2.8^‡^	0.011
Thrombus (%)	NA	43.2 ± 1.3	23.8 ± 1.4	7.1 ± 1.5^§^	< 0.001	44.9 ± 1.2	43.1 ± 1.3	20.2 ± 1.4	8.3 ± 1.5^§^	< 0.001
PLIA score	NA	1.90 ± 1.14^||^	1.18 ± 1.25^#^	1.01 ± 0.72	< 0.001	*N*A	0.89 ± 1.24^||^	0.67 ± 1.07^#^	0.64 ± 0.72	0.59

^*^p = 0.0003, ^†^*p* = 0.038, ^‡^*p* = 0.56, ^§^*p* = 0.76, ^||^*p* < 0.001, #*p* < 0.001, ^:^*p* = 0.001 (STEMI vs. stable-CAD)

**Fig. 4:**
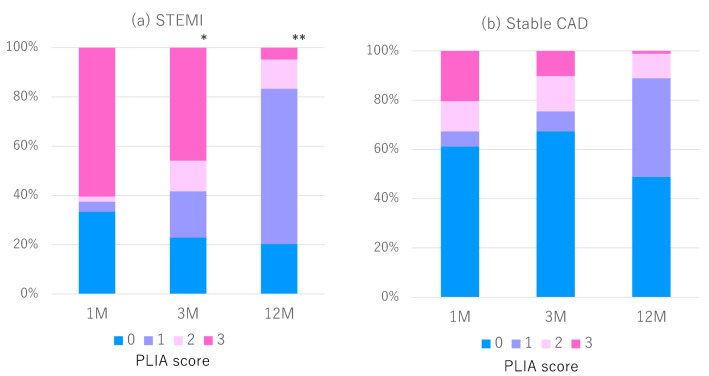
Incidence of PLIA score in the two pathogenetic groups: **a** the STEMI cohort: prevalence of high PLIA score (2 and 3) was significantly decreased during the follow-up period. *p* < 0.001* at 3 months, *p* < 0.001** at 12 months (reference 1 month). **b** The CAD cohort: *p* = 0.82 at 3 months, *p* = 0.12 at 12 months (reference 1 month)

The incidence of malapposed struts, incidence of thrombus, and IRP were, respectively, decreased during the follow-up period in the cohort (*p* < 0.001, respectively; for representative cases: Supplemental Fig. 1a). The average PLIA score was significantly decreased during follow-up period (p ≤ 0.001). Prevalence of high PLIA score (2 and 3) was also decreased during follow-up period (Fig. 4a).

### Primary and other OFDI endpoints of the stable-CAD cohort

OFDI measurements were serially compared between the immediate post-intervention timepoint and either 1- or 3-month follow-up, as shown in Table 3. In the stable-CAD cohorts, the %US significantly decreased between these timepoints (9.44 ± 0.78% to 7.78 ± 0.78%). The one at 1 month were approximately one half of the STEMI cohort. Progression of strut coverage was confirmed, while 12-month follow-up period (%US at 12 months: 1.07 ± 0.73%; *p* < 0.001; Fig. 3a). In parallel, the thickness of the neointima consistently increased during the 12 months of follow-up period as same as STEMI cohort. These data significantly and serially improved among these timepoints (representative cases: Supplemental Fig. 1b). Although, the average PLIA score and prevalence of high PLIA score (2 and 3) in the cohort were not significantly changed during follow-up period (*p* = 0.59, Fig. 4b).

### Comparing STEMI and stable-CAD cohorts

The %US at 1 month was significantly higher in the STEMI cohort than the stable-CAD cohort (18.75 ± 0.78% vs. 9.44 ± 0.78%; *p* < 0.001). However, that difference had decreased by 3 months (10.19 ± 0.77% vs. 7.78 ± 0.78%; *p* = 0.027) and had almost disappeared by 12 months (1.78 ± 0.73 vs. 1.07 ± 0.73; *p* = 0.45). The average NIT at 12 months was equivalent between the stable-CAD and STEMI cohorts. The average PLIA score at 1-month and 3 months in the STEMI cohort was significantly higher than that in the CAD cohort (*p* ≤ 0.001). However, the scores and its distribution in the STEMI cohort were markedly close to the stable-CAD cohort at 12-month follow-up period (Table 3 and Fig. 4).

### Clinical outcome at 12 months

Clinical endpoints at 12 months are shown in Table 4 and supplemental Table 2. The incidence of DOCE within 12 months was favorable in the two cohorts.

**Table 4:** Incidence of all-cause death and cardiac events at 12-month follow-up

	AMI cohort (*n* = 103)	Elective cohort (*n* = 101)
All-cause death	2 (1.9)	1 (1.0)
DOCE	7 (6.8)	2 (2.0)
Cardiac death	2 (1.9)	0 (0)
Target-vessel MI	4 (3.9)	0 (0)
Clinically driven TLR	5 (4.9)	2 (2.0)
Stent thrombosis	1 (1.0)	0 (0)

Patient-oriented composite endpoint: all-cause death, myocardial infarction, stroke and any revascularization, and DOCE device-oriented cardiac event

## Discussion

To the best of our knowledge, this study is the first prospective OFDI study to clarify the characteristics seen in patients with STEMI and stable-CAD in terms of early and late vascular healing after BP-SES implantation. The main results of this study are summarized as follows: first, remarkable progression of strut coverage was observed in both cohorts, especially in STEMI, in the early processes of vascular healing after BP-SES implantation, and these changes were consistent for 12 months. Second, the average NITs of the two cohorts were comparable at 12 months. Third, although elevated PLIA scores, considered a sign of vascular inflammation, were observed in the early phases of the STEMI cohort after BP-SES placement, they were significantly decreased during the follow-up period. Moreover, the prevalence of low PLIA scores in both cohorts was almost equivalent at the 12-month follow-up. Owing to the combination of these multifactorial improvements, qualitatively and quantitatively consistent neointimal stent coverage was achieved by the 12-month time point in both pathogenetic groups.

The clinical implications of this study are as follows: first, to provide novel information on temporal differences in the healing process between the different pathogeneses (stable-CAD and AMI) following the use of BP-SES; second, to allow a profound discussion regarding the optimal DAPT duration, by incorporating the mechanisms of vascular healing obtained from this imaging study into the accumulated evidence obtained from current and future event-based clinical studies; and third, to clarify the healing process, so that we will be able to concretely compare the results with other stents in future.

### BP-SES in patients with STEMI or stable-CAD

DES implantation in acute myocardial infarction has been identified as a major cause of delayed arterial healing [2]. Bioresorbable-polymer stents have the potential to resolve this problem in patients with STEMI. To our knowledge, no study has evaluated the quantitative and qualitative aspects of endovascular healing using OFDI within a short period of BP-SES implantation. A previous study, DISCOVERY1TO3, excluded patients with STEMI and did not qualitatively evaluate it [10]. The present study provides new information, being the first to demonstrate the performance of BP-SES, both quantitatively and qualitatively, in patients with STEMI. Regarding the early uncovered strut rate, we observed a rapid improvement from 1 to 3 months. The final uncovered rate was excellent, approaching zero at 12 months. We previously conducted the MECHANISM-AMI study using CoCr-EES [9]. Although not uniformly comparable, the uncovered strut rate after 3 months of BP-SES implantation was approximately 10%, higher than that of CoCr-EES at 5.5%. However, the uncovered strut rate and NIT in BP-SES improved rapidly and were remarkable, especially after 12 months of polymer absorption.

The difference in early healing between the stable-CAD and STEMI groups was evident: the stable-CAD cohort already demonstrated a low uncovered strut rate and a stable PLIA score after 1 month of implantation. The uncovered strut rate at 1 month post-implantation in the STEMI cohort was approximately twice that of the stable-CAD cohort (18.72% vs. 9.44%). There was an initial healing delay to a certain degree in the STEMI group compared with that in the stable-CAD group even after BP-SES implantation. However, the healing delay in the early phase was expected to catch up with that in the stable-CAD group once the polymer was absorbed and eliminated in the chronic phase.

Furthermore, interestingly, although the prevalence of regular thrombus showed a similar downward course in both cohorts, IRP decreased dramatically over time in the STEMI cohort (43.2% at 1 month to 7.1% at 12 months) compared with that in the stable-CAD cohort (4.4% at 1 month to 1.1% at 12 months), which had a lower incidence of IRP from the early point. Although IRP is controversial, it may also reflect the course of improvement in the instability of the vessel wall plaque in the STEMI cohort over time.

Moreover, the incidence of malapposed struts significantly decreased in both groups over time, whereas the malapposed area in the STEMI cohort increased to some extent in the early phase (Supplemental Table 3). We suspected that this unique phenomenon was caused by IRP and thrombus volume reduction in the malapposition area, in addition to the vessel wall.

In the present study, both cohorts of patients received DAPT for 1 year; the current guidelines for DAPT in patients with AMI recommend a duration of 3 months or longer. However, the appropriate duration of DAPT is also expected to vary between second- and third-generation DES. The MODEL-U trial with BP-SES demonstrated that 3 months of DAPT were feasible [11]. Since the MODEL-U trial included patients with AMI, DAPT for at least 3 months may be acceptable even in AMI. For third-generation stents, further studies are underway on P2Y12 inhibitor-based 1-month or 0 DAPT. Future interpretations based on a combination of imaging studies and large clinical trials will be necessary to establish the DAPT duration based on the characteristics of the stent as well as the pathology.

In summary, from a quantitative point of view, this study shows that although there are signs of early prolonged vascular healing in patients with STEMI compared with that in stable-CAD, such as (1) high initial % uncovered strut, (2) high frequency of IRP, and (3) mild increase in malapposed strut area, initial concerns about delayed arterial healing in AMI attenuate after polymer absorption over time and will mostly be overcome within 1 year.

### Pathological healing process and qualitative evaluation of healing after BP-SES

The PLIA score, which reflects qualitative healing, certainly demonstrated higher scores of 2 and 3 at 1 month in the STEMI cohort. This phenomenon, which shows a delayed healing process, suggested the impact of vessel wall characteristics on healing in patients with STEMI. However, subsequent NIT and PLIA scores showed steady improvement at 3 and 12 months. From this point of view, BP-SES brings good qualitative healing, in addition to superficial quantitative healing, even in patients with STEMI.

The most likely explanation for this phenomenon is the transformation of fibrin to mature neointimal tissue during the follow-up period. Moreover, we speculate that this specific phenomenon up to 3 months mainly reflects the vascular healing process after STEMI. We need to consider vascular responses for polymer absorption, which can potentially intensify local inflammation. However, the influence of this element might be considered minimal because of the lower PLIA score in the stable-CAD cohort. In fact, BP-SESs are designed, such that their polymers (poly DL-lactic acid) are absorbed over the course of 3–4 months. By the end of this period in the study, the polymer was completely dissolved, and the less inflammatory bare metal surface came into direct contact with the vascular wall, accommodating relatively healthy and homogeneous neointimal tissue growth for the remainder of our 12-month study period. Moreover, experimental data that validated the neointima after dual-polymer (DP)-EES and BP-SES implantation revealed that the number of cell tight junctions in the neointima after BP-SES implantation was significantly higher than that after DP-EES implantation. As a result, the authors concluded that the stability and robustness of the neointima was higher after BP-SES implantation [12].

The risk of a hypersensitive reaction caused by the remaining polymer is recognized as a major problem [2, 4, 5]. Inflammatory cell infiltration may lead to the destruction of the vascular wall structure. In first-generation permanent-polymer SESs, a phenomenon called peri-stent staining (PSS), marked by severe evagination [13], has been reported in some cases [14]. In addition, considering that late loss evaluated by QCA was very low with first-generation SESs, sirolimus was believed to suppress endovascular regeneration, and the interaction between the drug and polymer is believed to induce evagination. However, in the OFDI evaluation 12 months after BP-SES implantation, no threatening PSS was observed. Such improvement may result from a combination of rapid and complete polymer absorption and decreased sirolimus dose.

Furthermore, in a study that performed optical coherence tomography evaluation 9 months after BP-SES or DP-EES implantation, the proportion of uncovered struts was 1.02% for the former and 2.26% for the latter [15]. Moreover, Kuroda et al. reported that patients without PLIA on medium-term OCT experienced almost no late-phase TLR [8]. In the present study, the proportion of patients with a PLIA score of 0 or 1 at 12 months was approximately 80% in both cohorts. In summary, BP-SES significantly reduced the number of uncovered struts and showed high neointimal maturation (i.e., low PLIA scores). These positive results have the potential to solve the current problems of late catch-up and neo-atherosclerosis in durable polymer stents.

### Study limitations

This study has several limitations. First, the number of cases was relatively small. However, considering the extensive effort required for OFDI analysis, the sample size was as large as we could feasibly include. Second, no other type of stent was used as a control, as the groups were defined by disease condition. As a result, we could not directly address the difference between the BP-SES and other stents. Future studies should compare different types of stents. Third, the morphology and site of the lesion must be suitable for OFDI evaluation to proceed. This study consisted mostly of patients with multi-vessel disease, because OFDI assessments were scheduled during staged PCI. Finally, this study did not collect any long-term data. In the future, further study is needed to test for the existence of late catch-up and the appearance of evagination [16] after long-term observation. The present study had a limited follow-up period of only 1 year. If it is possible to continue OFDI several years after BP-SES implantation, it may be possible to confirm the features of BP-SESs in the long term.

## Conclusions

The strut-coverage and healing processes in the early phase after BP-SES implantation were significantly improved in both cohorts, especially markedly in STEMI patients. At 1 year, qualitatively and quantitatively consistent neointimal coverage was achieved in both pathogenetic groups.

## Supplementary Information

Below is the link to the electronic supplementary material.  

Supplemental Figure 1 (a): Representative cases of acute myocardial infarction. (b): Representative cases of stable coronary artery disease

Supplementary file2 (PDF 391 KB)

Supplementary file3 (DOCX 22 KB) Supplemental Tables 1-3: Supplemental Table 1: Medication at 12 months follow-up, Supp emental Table 2: Number of clinical events at 12 months follow-up, Supplemental Table 3: Difference in the malapposed area between he immediate postoperative and 1-month or 3-month postoperative measurements, as evaluated by multilevel analysis.
