# Supplementary material for: A serial optical frequency-domain imaging study of early and late vascular responses to bioresorbable-polymer sirolimus-eluting stents for the treatment of acute myocardial infarction and stable coronary artery disease patients: results of the MECHANISM-ULTIMASTER study
Source: Cardiovasc Interv Ther. 2021 Apr 25;37(2):281–92. doi: 10.1007/s12928-021-00777-4 (PMC8926965; doi:10.1007/s12928-021-00777-4)
Supplement: Supplementary file 1 — Supplemental Figure 1 (a): Representative cases of acute myocardial infarction. (b): Representative cases of stable coronary artery disease [file 12928_2021_777_MOESM1_ESM.pdf]

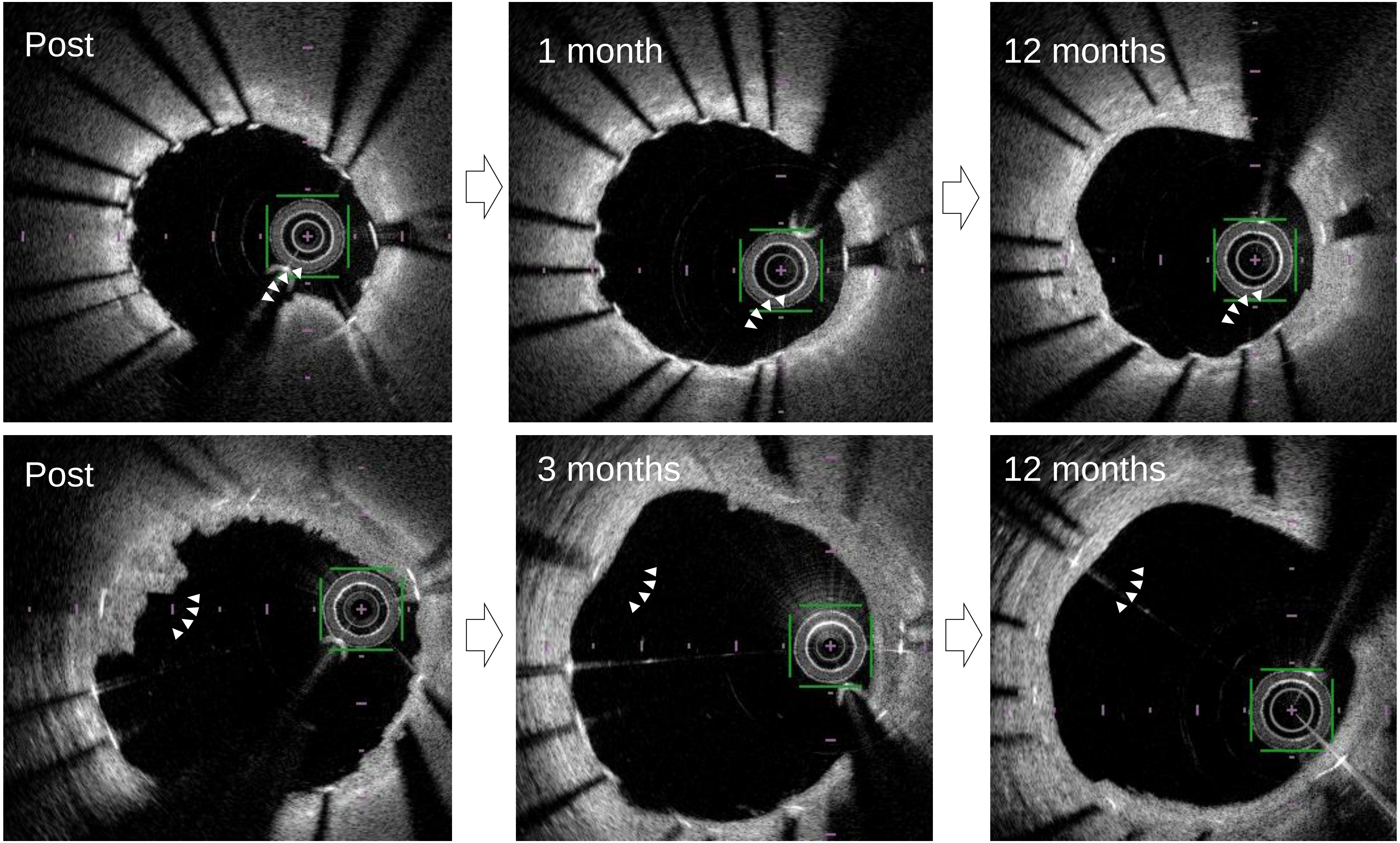

Supplemental Figure 1 (a)

Representative cases of STEMI

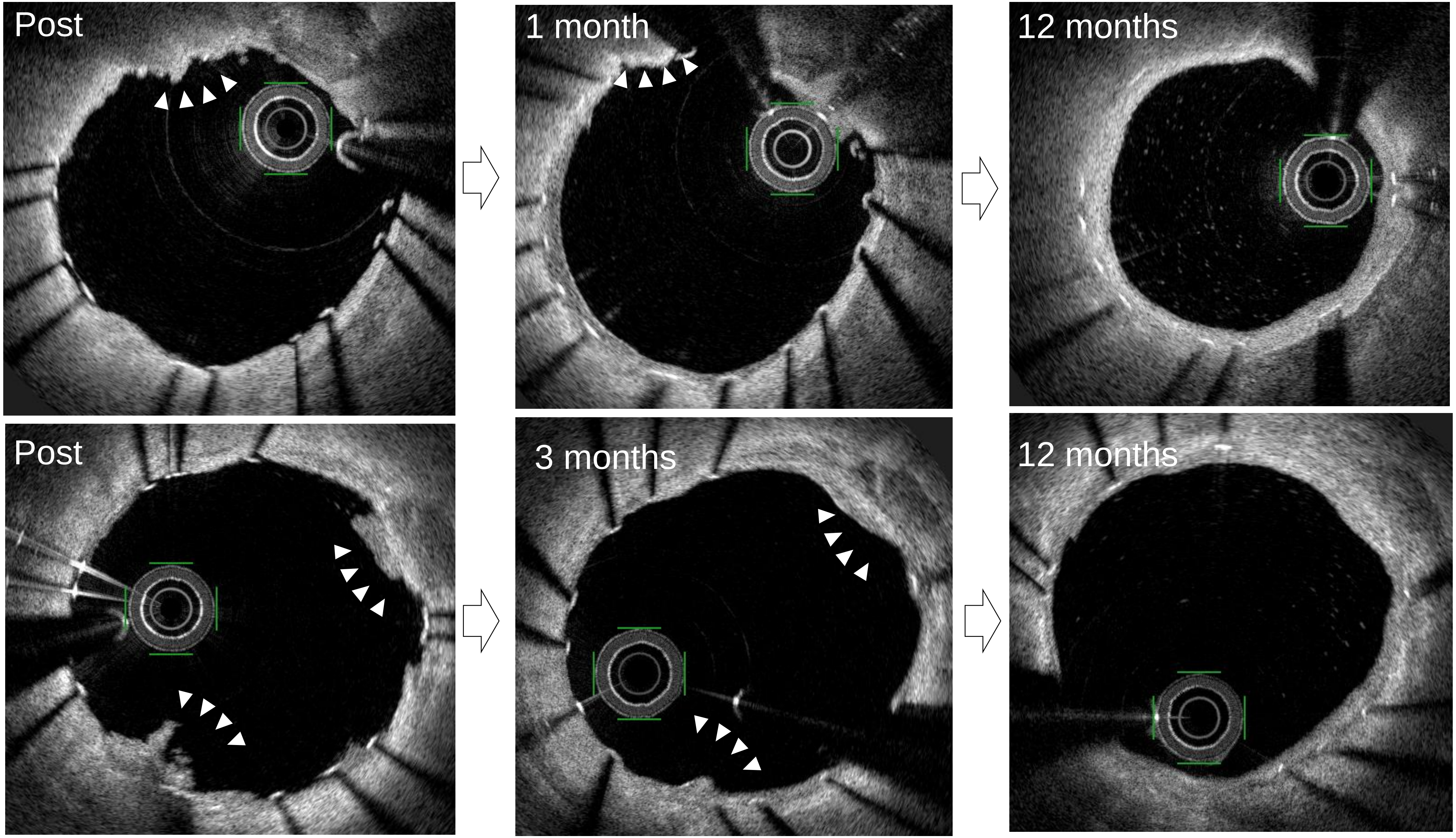

Supplemental Figure 1 (b)

Representative cases of stable CAD
