# Supplementary material for: A serial optical frequency-domain imaging study of early and late vascular responses to bioresorbable-polymer sirolimus-eluting stents for the treatment of acute myocardial infarction and stable coronary artery disease patients: results of the MECHANISM-ULTIMASTER study
Source: Cardiovasc Interv Ther. 2021 Apr 25;37(2):281–92. doi: 10.1007/s12928-021-00777-4 (PMC8926965; doi:10.1007/s12928-021-00777-4)
Supplement: Supplementary file 2 — Supplementary file2 (PDF 391 KB) [file 12928_2021_777_MOESM2_ESM.pdf]

Supplemental File

Study Protocol, result, and Core-laboratory reproducibility

A Serial Optical Frequency Domain Imaging Study of Early and Late Vascular Responses to Bioresorbable Polymer Sirolimus-eluting Stents for the Treatment of Acute Myocardial Infarction and Stable Coronary Artery Disease Patients -Results of the MECHANISM-ULTIMASTER study-

Tomonori Itoh[1], MD; Hiromasa Otake[2], MD; Takumi Kimura[1], MD; Yoshiro Tsukiyama[2], MD; Tatsuo Kikuchi[3], MD; Munenori Okubo[4], MD; Takatoshi Hayashi[5], MD, Ph. D, MBA; Takayuki Okamura[6], MD; Shoichi Kuramitsu[7], MD; Takashi Morita[8], MD; Shinjo Sonoda[9] MD, Shozo Ishihara[10] MD, Nehiro Kuriyama[11] MD, Takaaki Isshiki[12] MD, Tsunenari Soeda[13] MD, Kiyoshi Hibi[14] MD, Toshiro Shinke[15], MD; Yoshihiro Morino[1], MD;

On behalf of the MECHANISM-ULTIMASTER study group

1) Division of Cardiology, Department of Internal Medicine, Memorial Heart Centre, Iwate Medical University, Morioka Japan
2) Division of Cardiology, Department of Internal Medicine, Kobe University, Graduate School of Medicine, Kobe, Japan
3) Division of Cardiology, Edogawa Hospital, Edogawa, Tokyo, Japan
4) Department of Cardiovascular Medicine, Gifu Heart Centre, Gifu, Japan
5) Department of Cardiology, Hyogo Prefectural Awaji Medical Centre, Sumoto, Hyogo, Japan
6) Department of Medicine and Clinical Science, Yamaguchi University Graduate School of Medicine, Ube, Japan
7) Department of Cardiology, Kokura Memorial Hospital, Kitakyushu, Japan
8) Division of Cardiology, Osaka General Medical Centre, Osaka, Osaka, Japan
9) University of Occupational and Environmental Health, Kitakyusyu, Japan
10) Mimihara General Hospital, Sakai, Osaka, Japan
11) Miyazaki medical association hospital, Miyazaki, Miyazaki, Japan
12) Ageo Central General Hospital, Saitama, Japan
13) Nara Medical University, Nara, Japan
14) Division of Cardiology, Yokohama City University Medical Centre, Yokohama, Kanagawa, Japan
15) Division of Cardiology, Department of Internal Medicine, Showa University, Tokyo, Japan

The first two author equally contributed to this work.

Corresponding Author: Tomonori Itoh MD

Division of Cardiology, Department of Internal Medicine, Memorial Heart Centre, Iwate Medical University

2-1-1, Idai-Dori, Yahaba-Cho, Siwa-Gun, Iwate, 028-3695, JAPAN, e-mail: tomoitoh@iwate-med.ac.jp

MECHANISM-ULTIMASTER-AMI and MECHANISM-ULTIMASTER -Elective

- Exploring the **M**ECHANISM of early and late vascular responses to ULTIMASTER sirolimus-eluting stents for the treatment of ST-elevation **A**cute **M**yocardial **I**nfarction: principal investigator; Hiromasa Otake, MD
- **MECHANISM** of the pro-healing advantage of **ULTIMASTER** sirolimus-eluting stents assessed by early and late optical frequency domain imaging in **Elective** cases: principal investigator; Yoshihiro Morino, MD

These studies comply with the Declaration of Helsinki. The ethical committee of each participating institution approved the study protocols, and all patients provided written informed consent before inclusion.

Inclusion criteria

The patient inclusion criteria for the MECHANISM-ULTIMASTER-AMI study are as follows: AMI is defined according to the Third Universal Definition issued by the ESC/ACCF/AHA/WHF Task Force. The concrete definition is that cardiac troponin I or T rises or falls, with at least one value exceeding the 99th-percentile upper reference limit, and at least one of the following factors is also present: 1) ischaemic symptoms, 2) a new or expected significant ST-T change or a new left bundle branch block, 3) the appearance of an abnormal Q wave on electrocardiograms, 4) reduction of viable myocardium with

imaging evidence of new wall motion abnormality, 5) identification of an intracoronary thrombus by angiography or necropsy. The ECG diagnosis of STEMI was defined by the presence of ischaemic ST-segment elevation on more than two consecutive leads on ECG. The cut-off ST-altitude value was defined as >0.1 mV for all leads except V2 and V3 and as >0.2 mV in males or > 0.15 mV in females for V2 and V3.

The patient inclusion criteria for the MECHANISM-ULTIMASTER-Elective study were as follows: 1) patients must be good candidates for PCI for novel lesions using DES in the coronary arteries; 2) patients must be over 20 years and under 85 years of age at the time of enrolment; 3) patients must have provided written consent on their own behalf; 4) PCI treatment for other branch lesions must take place by OFDI 1 month or 3 months after initial stent placement, and the site must be observable; 5) the stent indwelling sites must be observable by OFDI 12 months after initial treatment.

Exclusion criteria

The exclusion criteria for the MECHANISM-ULTIMASTER-AMI study were defined as follows: 1) difficulty in completing 12 months of clinical follow-up, 2) no obvious ACS findings by coronary angiography (judgement by operator), 3) cardiogenic shock, 4) culprit lesion in the main trunk of the left coronary artery, 5) lesions with a reference

vessel diameter of less than 2.0 mm or more than 4.5 mm on visual inspection, 6) AMI-related artery at the site where a prior stent has already been placed, 7) chronic renal failure with a serum creatinine concentration of 2.0 mg/dl or higher at the time of admission, 8) current haemodialysis, 9) cancerous tumour accompanied by a life expectancy of 2 years or less, 10) plans to undergo surgery requiring withdrawal of antiplatelet agents within 3 months, 11) current pregnancy or plans to become pregnant, and 12) a history of drug side effects from aspirin or clopidogrel (not limited to patients for whom ticlopidine is confirmed to be safe).

The exclusion criteria for the MECHANISM-ULTIMASTER-Elective study were defined as follows: 1) difficulty in completing 12 months of clinical follow-up, 2) acute myocardial infarction or cardiogenic shock, 3) heart failure, 4) lesions of the main trunk of the left coronary artery, 5) reference vessel diameter of less than 2.0 mm or more than 4.5 mm, 6) in-stent restenosis, 7) chronic renal failure (serum creatinine ≥2.0 mg/dl), 8) haemodialysis, 9) cancer accompanied by a life expectancy of less than 2 years, 10) elective surgeries requiring withdrawal of dual antiplatelet therapy, 11) current or planned pregnancy, 12) a prior history of allergies to aspirin or clopidogrel, and 13) being judged inappropriate by investigators for any other reason.

The patient in- and exclusion criteria for the both studies are described in of details on the

UMIN CTR web page (https://upload.umin.ac.jp/cgi-open-bin/ctr/ctr_view.cgi?recptno=R000024774, https://upload.umin.ac.jp/cgi-open-bin/ctr/ctr_view.cgi?recptno=R000023994).

Definition of clinical characteristics

Patient background characteristics were collected through an on-site registration system. Coronary risk factors were defined as hypertension, diabetes mellitus, dyslipidaemia, current smoking habits, a family history of coronary artery disease, and chronic renal failure. Diabetes mellitus was defined as blood glucose levels ≥ 200 mg/dl two hours after an oral glucose tolerance test, blood glucose levels ≥ 200 mg/dl, fasting glucose levels >126 mg/dl, or the presence of a prior clinical diagnosis. Dyslipidaemia was defined as total cholesterol levels ≥ 240 mg/dl, low-density lipoprotein cholesterol (LDL-C) levels ≥ 140 mg/dl, high-density lipoprotein cholesterol (HDL-C) levels < 40 mg/dl, or a statin prescription. Renal insufficiency was evaluated by the estimated glomerular filtration rate (e-GFR) (for Japanese patients; Japan Kidney Association). The terminal stage of renal failure was defined as e-GFR＜30 ml/min/1.73 mm^2, and chronic kidney disease was defined as e-GFR＜60 ml/min/1.73 mm^2.

PCI procedure, OFDI acquisition at index PCI and follow-up

At the beginning of the PCI procedure, all patients were injected with a heparin bolus.

The PCI procedure and OFDI acquisition were performed according to Judkin's technique via the trans-radial or femoral approach using a >=6-French guiding catheter system. After the insertion of a 0.36 mm intervention guide wire, balloon angioplasty was performed for the target lesion, and a BP-SES was deployed to the culprit lesion. Culprit lesions were treated with either one or two BP-SES. If necessary, the use of two stents with subsequent balloon dilatation was approved. After optimal stent implantation was achieved, the final OFDI acquisition was performed.

The imaging catheter was carefully advanced distal to the target lesion under fluoroscopic guidance. Motorized pullback OFDI acquisition was performed at a pullback rate of 20 mm/sec or 40 mm/sec throughout the stented segment, with a margin segment ≥ 5 mm from the culprit lesion. Contrast medium was flushed continuously through the guiding catheter during image acquisition. Before OFDI acquisition, intracoronary administration of 0.5 mg of nitroglycerine was performed before and after stent deployment. Lactated Ringer's solution or low-weight dextran solution was available if necessitated by patients' clinical condition.

At 1-month, 3-month and 12-month follow-up, coronary angiography and OFDI were

performed according to a standard procedure using a ≥5-French catheter. The precise parameters were set determined by the rules of the QCA core laboratory.

Quantitative coronary angiography (QCA)

The target lesion was analysed using a QCA system (Q angio) using the external diameter of the contrast-filled guiding catheter as the calibration standard. Minimal lumen area, lesion length, percent diameter stenosis, and reference diameter were measured. Percent diameter stenosis was calculated from the minimal lumen diameter and the reference diameter. All QCA data were evaluated at Iwate Core Analysis Laboratory (ICAL) as the central core laboratory.

Supplemental definition of OFDI image and image analysis of Core-lab

The definition of intra-stent tissue was defined as follows: Smooth protrusion was the bowing of the plaque between stent struts without disruption. Disrupted fibrous-tissue protrusion was defined as the disruption of underlying fibrous tissue protruding between stent struts into the lumen. Irregular protrusion was defined as protrusion of material with an irregular surface into the lumen between stent struts (>0.25 mm). A thrombus was defined as a mass attached of the luminal surface of a stent strut or floating within the

lumen (>0.25 mm).

Standard operation procedure for OFDI analysis was set up between the both core-labs before starting studies. All OFDI measurements were confirmed by multiple reviewers in each core laboratory and discussed any questionable images until a consensus was reached. Moreover, remained images were discussed until a consensus was reached between the both core laboratories.

Dual antiplatelet therapy

Patients who had not taken antiplatelet therapy beforehand were loaded with aspirin 81-200 mg and prasugrel 20 mg or clopidogrel 300 mg as early as possible before PCI. In this study, we recommended a combination of aspirin and thienopyridine (prasugrel or clopidogrel) for 12 months (DAPT) according to the guidelines for acute coronary syndrome as well as those for stable angina pectoris. If aspirin or thienopyridine needed to be replaced by another medicine due to a drug allergy or other circumstances, we recommended continued administration for as long as possible up to 12 months.

Endpoints

Composite endpoints: The patient-oriented composite endpoint was defined to include

all-cause death, any MI including non-target territory, any repeat revascularization, and stroke as of the 12-month follow-up. Then, to compare device performance in the two cohorts, we defined device-oriented cardiac events (DOCEs), including cardiac death, target vessel MI and clinically driven TLR, as another endpoint.

Statistical analysis

Rationale for the number of cases: MECHANISM-ULTIMASTER-Elective

In the DISCOVERY 1TO 3 trial previously conducted in Europe, the average covered strut ratio (CSR) after 3 months of ULTIMASTER implantation was 95.7%, and the proportion of patients with CSR of 90% or more was 88% of the total. In the present study, it was assumed that the results would be equivalent, and the primary endpoint value was assumed to be 88%.

In this study, the number of required cases was calculated under the following conditions, with the expectation that the primary endpoint would be 88% or more on clinical examination.

$N=(Z\ power\sqrt{\ }p(1-p)+z1-\alpha\sqrt{\ }p0(1-p0))/p-p0)^2$

H 0: patient proportion with ULTIMASTER-CSR> 90% = 73%; HA: percentage of patients with ULTIMASTER-CSR> 90% = 88%, threshold CSR ratio (P 0) = 73%,

expected CSR ratio (P) = 88%, significance level = 5% (one-sided test), power = 80%.

The number of cases, N, was calculated under this condition using the sample size formula (below) for testing the ratio of one group ($Z1 - \alpha = 1.64$ because the one-sided significance level is 5%, Z power = 0.841 from the power of 80%).

Based on the above, the number of cases required for this verification was calculated to be 45 cases, and we decided to register 50 cases to allow for dropouts.

Rationale for the number of cases: MECHANISM-ULTIMASTER-AMI

We considered that the one-month CSR of the DISCOVERY 1TO3 trial conducted in the past was 85.8 ± 11.2% and that the DISCOVERY trial included elective cases. Therefore, we assumed that the true value of CSR one month after the primary endpoint was 80% with an SD of 12%. From the above, since the CSR after 2 weeks of the MECHANISM-AMI test using the EES was 80%, this value was set as the performance target of ULTIMASTER's CSR. To prove that ULTIMASTER's CSR is non-inferior to that of the EES, we calculated the number of cases as below, assuming a clinical non-inferiority margin of 5%. Based on this assumption, the required number of cases was calculated to be 38 cases. We decided to register 50 cases because we expected a high dropout rate due to the special situation of STEMI and because clear image acquisition

was needed for analysis. For the observation of CSR changes at three months, the size of the three-month group was set to 50 cases, and the same was done for the one-month group.

H_0: ULTIMASTER□-CSR ≤ 75%; H_A: ULTIMASTER□-CSR > 75%; Alpha = 0.05 (one-sided test); CSR performance target = 80%; true CSR value of ULTIMASTER® = 80%, standard deviation of ULTIMASTER® CSR = 12%, power = 80%.

Core-laboratories reproducibility

Inter- and intra-observer reproducibility for the covered/uncovered and PLIA

	Cover/Uncovered	PLIA score
MECHANISM-UM-Elective (ICAL)	Inter-: 0.97<br>Intra-: 0.97	Inter-: 0.97<br>Intra-: 0.98
MECHANISM-UM-AMI (KCCL)	Inter-: 0.69<br>Intra-: 0.92	Inter-: 0.88<br>Intra-: 0.91

PLIA data of KCCL was published in EuroIntervention 2019;14:1751-1759

Kappa index between the two core-laboratories

	Cover/Uncovered	PLIA score
Inter-core laboratory	0.80	0.97

Incidence of staged PCI and Follow up CAG+OFDI

		Total Number of Early Follow up	Number of OFDI with follow-up CAG	Number of OFDI for registered vessel with staged PCI for another vessel
STEMI	1M	48	2	46
	3M	51	16	35
Stable-CAD	1M	47	3	44
	3M	49	13	36

Number of corrective measures based on the first post-procedural OFDI run

		Total Number of OFDI at index PCI	Number of finished cases based on the first post-procedure OFDI run	Number of corrective measures based on the first post-procedural OFDI run
STEMI	1M	50	45	5*
	3M	48	42	6**
Stable-CAD	1M	48	32	16*
	3M	51	34	17**

There were no significant differences between the two groups. *: p=0.28, **: p=0.24

Table 1. (supplemental legend)

There was no difference in patient characteristics between the two cohorts except that prior myocardial infarction and PCI were more frequent in the stable-CAD cohort and smoking was more frequent in the STEMI cohort. Postprocedural QCA analysis results in the two cohorts were comparable, except that the stent diameter was larger in the STEMI cohort than in the stable-CAD cohort and that stenosis as a percentage of diameter was slightly higher in the former cohort than in the latter.

Appendix: Institutes and co-investigators participating in the study

MECHANISM-ULTIMASTER-AMI and MECHANISM-ULTIMASTER-Elective study group

- Yudai Shimoda, MD – Division of Cardiology, Department of Internal Medicine, Memorial Heart Centre, Iwate Medical University
- Takumi Inoue, MD – Hyogo Prefectural Awaji Medical Centre, Sumoto, Hyogo, Japan
- Hiroyuki Okura, MD; Tsunenari Soeda, MD – Nara Medical University
- Toru Kataoka, MD. Tomokazu Iguchi, MD – Bell Land General Hospital, Sakai, Osaka, Japan

- Takashi Takenaka, MD – National Hospital Organization Hokkaido Medical Centre, Sapporo, Japan
- Masaki Sakakibara, MD; Yasushi Jinno, MD – Handa City Hospital, Handa, Aichi, Japan
- Yoshinori Yasaka, MD; Tomofumi Takaya, MD – Hyogo Brain and Heart Centre, Himeji, Hyogo, Japan
- Junya Shite, MD; Amane Kozuki, MD – Osaka Saiseikai Nakatsu Hospital, Osaka, Osaka, Japan
- Makoto Kadotani, MD – Yasuhiro Kaetsu, MD; Kakogawa Central City Hospital, Kakogawa, Hyogo, Japan
- Yoshitomo Tsutsui, MD – Saiseikai Fukuoka General Hospital

MECHANISM-ULTIMASTER-Elective

Clinical Event Committee: Kengo Tanabe MD (Mitsui Memorial Hospital), Hideki Ishii MD (Nagoya University Hospital)

Clinical Safety Committee: Ken Kozuma MD (Teikyo University Hospital), Yoritaka Otsuka MD (Fukuoka-wajiro Hospital)

Statistician: Mitsuru Abe MD (National Kyoto Medical Centre)

MECHANISM-ULTIMASTER-AMI

Clinical Event Committee: Ken Kozuma MD (Teikyo University Hospital), Kengo Tanabe MD (Mitsui Memorial Hospital)

Clinical Safety Committee: Yoritaka Otsuka MD (Fukuoka-wajiro Hospital), Gaku Nakazawa MD (Tokai University Hospital)

Statistician: Mitsuru Abe MD (National Kyoto Medical Centre)

Supplemental Figure legend

Supplemental Figure 1: Representative cases from the present study: (a) STEMI cohort, (b) stable-CAD cohort

Supplemental Figure 2: PLIA scores measured from OFDI at the 1-month (a), 3-month (b), and 12-month follow-ups (c)

Reference for studies (discussion section)

DESSOLVE III study

de Winter RJ, Katagiri Y, Asano T, Milewski KP, Lurz P, Buszman P, Jessurun GAJ, Koch KT, Troquay RPT, Hamer BJB, Ophuis TO, Wohrle J, Wyderka R, Cayla G, Hofma SH, Levesque S, Zurakowski A, Fischer D, Kosmider M, Goube P, Arkenbout EK, Noutsias M, Ferrari MW, Onuma Y, Wijns W, Serruys PW. A sirolimus-eluting bioabsorbable polymer-coated stent (MiStent) versus an everolimus-eluting durable polymer stent (Xience) after percutaneous coronary intervention (DESSOLVE III): a randomised, single-blind, multicentre, non-inferiority, phase 3 trial. *Lancet.* 2018;391:431-40.

CENTURY II trial

Wohrle J, Markovic S, Rottbauer W, Muramatsu T, Kadota K, Vazquez-Gonzalez N, Odenstedt J, Serra A, Antoniucci D, Varenne O, Saito S, Wijns W. Bioresorbable polymer sirolimus-eluting coronary stent compared with permanent polymer everolimus-eluting coronary stent implantation for treatment of small vessel coronary artery disease: CENTURY II trial. *EuroIntervention.* 2016;12:e167-74.

BIOFLOW V study

Kandzari DE, Koolen JJ, Doros G, Massaro JJ, Garcia-Garcia HM, Bennett J, Roguin A, Gharib EG, Cutlip DE, Waksman R. Ultrathin Bioresorbable Polymer Sirolimus-Eluting Stents Versus Thin Durable Polymer Everolimus-Eluting Stents. *J Am Coll Cardiol.* 2018;72:3287-97.
