# Supplementary material for: A serial optical frequency-domain imaging study of early and late vascular responses to bioresorbable-polymer sirolimus-eluting stents for the treatment of acute myocardial infarction and stable coronary artery disease patients: results of the MECHANISM-ULTIMASTER study
Source: Cardiovasc Interv Ther. 2021 Apr 25;37(2):281–92. doi: 10.1007/s12928-021-00777-4 (PMC8926965; doi:10.1007/s12928-021-00777-4)
Supplement: Supplementary file 3 — Supplementary file3 (DOCX 22 KB) Supplemental Tables 1-3: Supplemental Table 1: Medication at 12 months follow-up, Supp emental Table 2: Number of clinical events at 12 months follow-up, Supplemental Table 3: Difference in the malapposed area between he immediate postoperative and 1-month or 3-month postoperative measurements, as evaluated by multilevel analysis. [file 12928_2021_777_MOESM3_ESM.docx]

| **Supplemental Table 1: Medication at 12 months follow-up** | | |
| --- | --- | --- |
|  | STEMI (n=101) | Stable-CAD (n=97) |
| DAPT, n (%) | 96 (95.0) | 87 (89.7) |
| Aspirin, n (%) | 97 (96.0) | 95 (97.9) |
| Thienopyridine, n (%) | 100 (99) | 90 (92.8) |
| Statins, n (%) | 98 (97.0) | 86 (88.7) |
| ACE/ARB, n (%) | 82 (81.2) | 55 (56.7) |
| β-blockers, n (%) | 83 (82.2) | 49 (50.5) |

Patients with death and unknown were excluded (STEMI death 2; Stable-CAD unknown 3, death 1)

**Supplemental Table 2: Number of clinical events at 12 months follow-up**

|  | STEMI cohort (n=103) | Stable-CAD cohort (n=101) |
| --- | --- | --- |
| All-cause death | 2 (1.9) | 1 (1.0) |
| Cardiac death | 2 (1.9) | 0 (0) |
| Any MI | 7 (6.8) | 0 (0) |
| Target-vessel MI | 4 (3.9) | 0 (0) |
| Definite/probable stent thrombosis | 1 (1.0) | 0 (0) |
| Major bleeding | 1 (1.0) | 0 (0) |
| Any TLR | 7 (6.8) | 5 (5.0) |
| Clinical driven TLR | 5 (4.9) | 2 (2.0) |
| Any TVR | 19 (18.4) | 6 (5.9) |
| Any revascularization | 21 (20.4) | 8 (7.9) |
| Angiographic binary restenosis | 6 (5.8) | 5 (5.0) |
| Patient-oriented composite endpoint | 23 (22.3) | 9 (8.9) |
| DOCE | 7 (6.8) | 2 (2.0) |

TLR: target lesion revascularization; MI: myocardial infarction

Patient-oriented composite endpoint: all-cause death, myocardial infarction, stroke and any revascularization, DOCE (device-oriented cardiac event)

**Supplemental Table 3:** Difference in the malapposed area between the immediate postoperative and 1-month or 3-month postoperative measurements, as evaluated by multilevel analysis

|  | STEMI | | | | stable CAD | | | |
| --- | --- | --- | --- | --- | --- | --- | --- | --- |
|  | Estimate  (mm^2^) | SE | 95%CI | p-value | Estimate  (mm^2^) | SE | 95%CI | ｐ-value |
| 1M－Post | 0.02748 | 0.01399 | 0.00005 ～ 0.05491 | 0.0496 | 0.01199 | 0.0139 | -0.01525 ～ 0.03923 | 0.3884 |
| 3M－Post | 0.00706 | 0.0132 | -0.03294 ～ 0.01882 | 0.593 | -0.03241 | 0.01421 | -0.06027 ～ -0.004550 | 0.0226 |
